# Associations between dietary factors and morbidity burden in occupational radiation workers: a cross-sectional study in Guangzhou, China

**DOI:** 10.3389/fnut.2026.1808021

**Published:** 2026-07-31

**Authors:** Hai-bo Huang, Yan Zhang, Yi-wei Su, Shi-feng Hou, Qiang-qiang Wang, Si-qi Tang, Peng Rao, Zhi Wang, Jian-yu Wang

**Affiliations:** Key Laboratory of Occupational Environment and Health, Guangzhou Twelfth People's Hospital, Guangzhou, China

**Keywords:** China, cross-sectional, dietary determinants, morbidity burden, occupational radiation

## Abstract

**Objective:**

This cross-sectional study aimed to investigate the associations between dietary factors and morbidity burden among occupational radiation workers in Guangzhou, China, a population with chronic low-dose radiation exposure.

**Methods:**

From January to December 2024, 1,080 medical radiation workers were enrolled. Dietary intake was assessed using a validated food frequency questionnaire, and morbidity burden was defined as the presence of clinically diagnosed chronic conditions. Multivariable logistic regression models were employed to evaluate associations, adjusting for demographic, occupational, and lifestyle confounders.

**Results:**

The overall morbidity prevalence was 45.5%, with thyroid diseases being most common (23.5%). Significant dietary differences were observed: higher tea consumption (≥3 times/week: OR 0.41, 95% CI 0.26 ~ 0.64), vegetable intake (≥3 times/week: OR 0.44, 95% CI 0.29 ~ 0.67), and fruit consumption (≥200 g/week: OR 0.71, 95% CI 0.53 ~ 0.95) were associated with lower morbidity odds. In contrast, high red meat intake (≥3 times/week: OR 1.88, 95% CI 1.34 ~ 2.67) and a preference for meat over vegetables were associated with higher morbidity odds.

**Conclusion:**

Specific dietary patterns, particularly plant-based foods and tea, are inversely associated with morbidity burden in radiation workers. These findings support integrating tailored nutritional guidelines into occupational health frameworks to improve health outcomes among radiation workers.

## Introduction

Occupational radiation workers face distinct health challenges stemming from chronic exposure to ionizing radiation, a Group 1 carcinogen as classified by the International Agency for Research on Cancer (1). Although stringent protective measures are widely implemented to limit external exposure, concerns persist regarding the long-term effects of cumulative low-dose radiation (2). Evidence indicates that medical radiation workers experience elevated risks of both malignant and non-malignant diseases (3). For example, studies among medical X-ray workers report significantly increased relative risks for leukemia, esophageal cancer, and ischemic heart disease (4). These observations underscore a multifactorial morbidity burden linked to prolonged radiation exposure, encompassing not only cancer but also cardiovascular, hematopoietic, and metabolic disorders (5).

The underlying mechanisms involve disruption of critical physiological pathways. Ionizing radiation induces DNA damage, oxidative stress, and systemic metabolic dysregulation (6). Nutrient metabolism proves especially susceptible: radiation exposure alters protein turnover, often leading to negative nitrogen balance; promotes lipid peroxidation and hyperlipidemia (7); and depletes endogenous antioxidants such as vitamins C and E (8). Resulting metabolic disturbances may exacerbate chronic conditions, including cardiovascular diseases and immune dysfunction, highlighting the potential value of nutritional strategies in mitigating radiation-related harm (9).

Certain dietary components possess antioxidant, anti-inflammatory, or DNA-repair-promoting properties that may counteract radiation-induced damage (10). Animal and clinical studies suggest that high-quality protein, unsaturated fatty acids, and antioxidant-rich fruits and vegetables can enhance physiological resilience (11, 12). Nevertheless, existing research largely concentrates on isolated nutrients or experimental models of acute high-dose exposure (13). Population-based evidence connecting holistic dietary patterns with the overall morbidity burden among radiation workers remains limited (14). This represents a significant gap, given the need for practical, integrated dietary guidelines tailored to real-world occupational health scenarios.

Epidemiological studies on radiation workers have traditionally emphasized cancer-specific risks or mortality, frequently overlooking non-malignant conditions and composite health outcomes (15). To date, no investigation has systematically examined the association between dietary factors and multidimensional health status in this occupational group (16). Guangzhou, a major center for medical and industrial radiography in southern China, offers an ideal setting to address this knowledge gap, owing to its high density of radiation facilities and diverse workforce (17).

This cross-sectional study therefore aims to investigate the relationship between dietary patterns and morbidity burden among occupational radiation workers in Guangzhou. It seeks to characterize prevalent dietary habits and evaluate their associations with a spectrum of health conditions (18). By identifying dietary determinants that may alleviate the health impacts of chronic radiation exposure, the findings are expected to inform targeted nutritional guidelines and support the integration of dietary management into occupational health frameworks for this vulnerable population.

## Methods

### Study design and participant recruitment

This cross-sectional study was designed to systematically examine the associations between dietary patterns and morbidity outcomes among radiation workers in Guangzhou, China, with a focus on ensuring methodological transparency and reproducibility. Data collection was conducted from January to December 2024 at Guangzhou Twelfth People’s Hospital, a regional center for occupational health surveillance. The study cohort was derived from the hospital’s occupational health registry, which initially identified approximately 1,300 eligible individuals. Recruitment was conducted under the framework of a national occupational health surveillance program. Official administrative notifications were sent to relevant institutions to invite eligible workers for a comprehensive health evaluation. After applying predefined eligibility criteria, 1,103 participants voluntarily provided written informed consent and were enrolled, forming a heterogeneous group of medical radiation professionals engaged in diagnostic imaging, radiation therapy, and interventional radiology.

**Figure 1:**
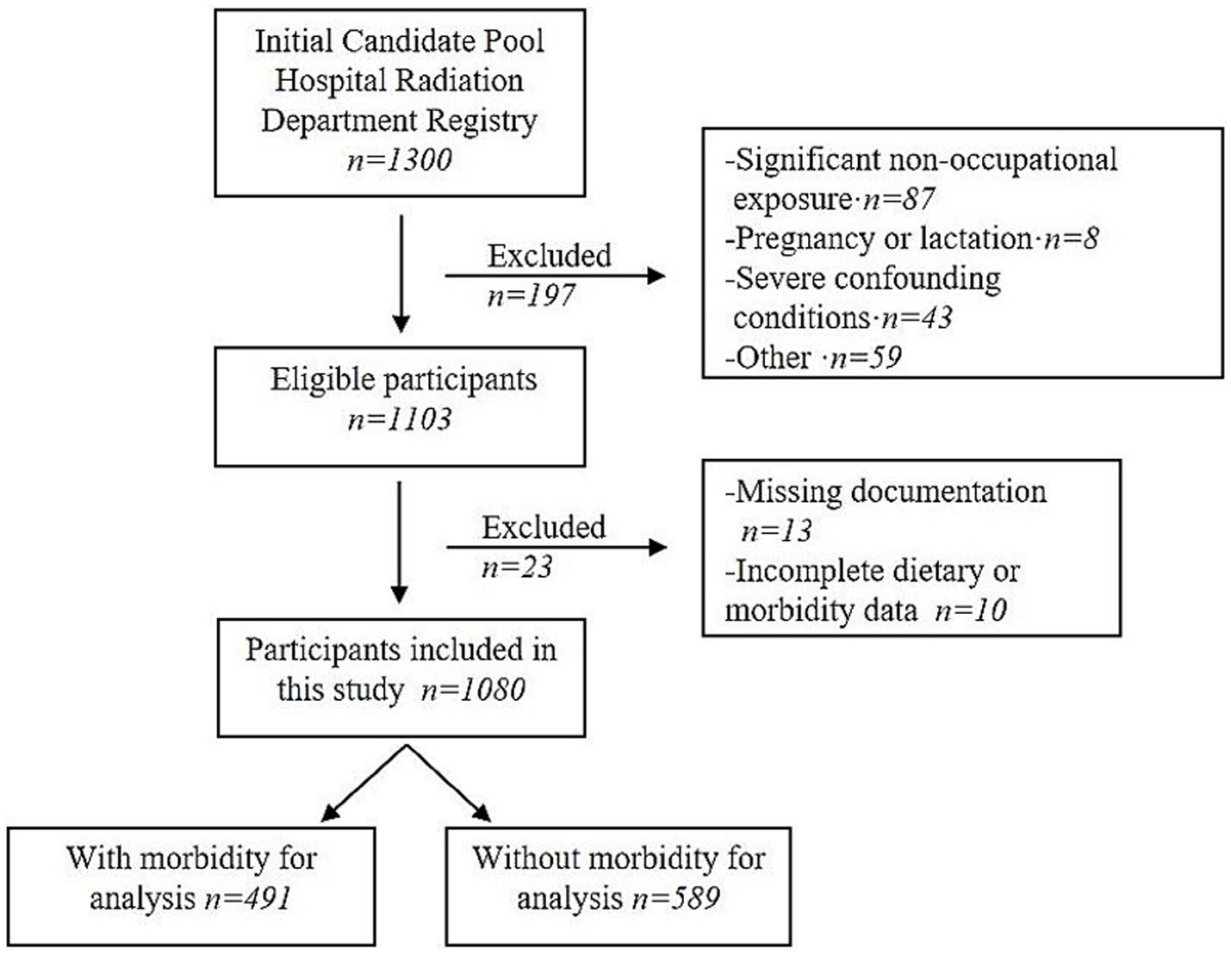
Flow chart of selecting included participants.

Inclusion criteria required participants to: (1) hold full-time positions in medical radiation fields with verified occupational exposure records for ≥1 year; (2) be aged ≥18 years; and (3) have completed a comprehensive dietary assessment and clinical evaluation during the study period. Following data quality checks, 23 participants were excluded due to incomplete or missing essential data, resulting in a final analytical sample of 1,080 participants. Selecting of participants is showed in Figure 1.

Exclusion criteria encompassed: (1) significant non-occupational radiation exposure; (2) incomplete dietary or morbidity data; (3) pregnancy or lactation; and (4) severe conditions unrelated to radiation exposure that could confound morbidity assessments.

The study protocol was approved by the Ethics Committee of Guangzhou Twelfth People’s Hospital (Approval No. 2024021). All participants provided written informed consent. To enhance data accuracy, occupational histories and clinical parameters were cross-verified through electronic health records and structured face-to-face interviews.

### Sample size

The sample size for this cross-sectional study was determined using the formula for estimating a population proportion in cross-sectional study (19):


$$n=\frac{{Z^{2}}_{1-\alpha /2}\times p\times (1-p)}{\varepsilon^{2}}$$

Here: *Z*_1-α/2_ = 1.96 (*Z*-score for 95% confidence level, *α* = 0.05), p represents the anticipated prevalence of the primary outcome, and *ε* denotes the margin of error. Based on prior regional studies, the prevalence of elevated morbidity burden among occupational radiation workers was estimated at 35% (*p* = 0.35). With ε set at 0.03, the calculation yielded a minimum sample size of 972 participants.

To address potential non-response or missing data, the target sample size was inflated by 15%, resulting in a final target of approximately 1,120 participants. This adjustment aligned with the annual cohort of eligible radiation workers undergoing health examinations at Guangzhou Twelfth People’s Hospital during the 2024 study period. The final analyzed sample of 1,080 participants met the minimum sample size requirement.

### Dietary assessment

Habitual dietary intake was assessed using a structured, semi-quantitative food frequency questionnaire (FFQ) standardized and validated by the National Occupational Health Surveillance Program for radiation workers. The assessment was conducted through standardized face-to-face interviews by trained investigators during the participants’ scheduled physical examinations at the hospital January and December 2024. As participants arrived in batches, they were guided to a private interview area where investigators recorded their responses on standardized paper questionnaires. Based on the pre-defined options in the original instrument, participants reported the intake frequency of seven key dietary items linked to antioxidant status and radioprotective potential: tea, coffee, seafood, red meat, vegetables, and fruits. To minimize recall bias, the questionnaire referenced typical weekly consumption, and investigators provided visual aids (e.g., portion size photographs) to help standardize responses. All paper records were subsequently double-entered into EpiData version 3.1 (EpiData Association, Odense, Denmark) by two independent researchers to ensure data accuracy and minimize transcription errors.

The consumption categories were standardized according to the original questionnaire design to facilitate rapid assessment in a high-volume occupational screening setting:

- Tea and coffee: “never,” “occasionally (<3 times/week),” or “frequently (≥3 times/week).”
- Seafood, meat, and vegetables: categorized into three levels: “none,” “<3 times/week,” and “≥3 times/week.”
- Fruit intake: classified as “none,” “<200 g/week,” and “≥200 g/week.”

Given the unbalanced distribution of participants across intake categories, the two lowest frequency groups for seafood, red meat, vegetables, and fruit were merged into a single reference category (e.g., “0 ~ 2 times/week”) prior to logistic regression analysis to ensure adequate statistical stability of the reference group. Additional dietary behaviors (taste preferences, dietary balance) were recorded to contextualize dietary patterns within lifestyle factors.

### Occupational health status assessment

Health status was evaluated through a multimethod framework integrating self-reported data and objective clinical measures. The self-reported component was included in the same structured, paper-based questionnaire as the FFQ and was completed during the same face-to-face interview session. Data collection included:

- Demographics and lifestyles: Age, sex, BMI, education, smoking status, alcohol use, physical activity (assessed via validated categorical scales).
- Occupational histories: Years of service, daily working hours, profession, position responsibilities, academic title, and in-room operation status.

Clinical measurements, including physical examinations and laboratory tests, were conducted by certified medical staff at the hospital’s specialized occupational health center. This multidimensional approach ensured comprehensive capture of covariates potentially influencing morbidity outcomes.

### Morbidity burden assessment

Morbidity burden was operationalized as the presence of clinically diagnosed chronic conditions, classified according to ICD-10 codes. To ensure diagnostic accuracy, morbidity status was determined based on a combination of self-reported medical history and, more importantly, verified clinical diagnoses provided by physicians during the physical examination. All morbidity data were extracted and cross-referenced with the hospital’s Electronic Health Records (EHR) to ensure that the findings were based on professional medical evaluations rather than self-report alone. Conditions included: Thyroid diseases (E00-E07), metabolic disorders (E70-E90), respiratory diseases (J00-J99), digestive conditions (K00-K93), cardiovascular diseases (I00-I99), lens opacity (H25-H28), and neoplasms (C00-D49). Each category was defined by specific diagnoses (e.g., hypothyroidism E03.9, hypertension I10) to standardize outcomes and enhance comparability.

### Statistical analysis

The statistical analyses were performed using R software version 4.4.1 (R Foundation for Statistical Computing, Vienna, Austria). Descriptive statistics were computed for all variables. Continuous variables that followed a normal distribution are summarized as mean ± standard deviation, and comparisons between groups were made using Student’s t-tests. For continuous variables that deviated significantly from a normal distribution, data are presented as median (interquartile range), and the Mann–Whitney *U* test (for two-group comparisons) or the Kruskal–Wallis test (for multi-group comparisons) was utilized. Categorical variables are expressed as counts and percentages, and associations were evaluated using Pearson’s chi-square test or Fisher’s exact test, as appropriate for the data.

The primary analysis employed binary logistic regression to assess the associations between dietary factors and morbidity burden, with results reported as odds ratios (ORs) alongside their 95% confidence intervals (CIs). This method was chosen for its suitability in modeling a binary outcome. The analysis followed a sequential approach through three tiers of adjustment: a crude model with no adjustments, Model 1 adjusted for age and sex, and Model 2, which included further adjustments for race, BMI, years of radiological service, daily working, education background, professions, position responsibilities, academic title, in-room operation, active smoking status, alcohol consumption status, physical exercise status, daily sedentary time, frequency of staying up late and sleep duration.

In these models, all dietary factors and dietary preferences were analyzed as categorical variables based on their predefined frequency and preference groups. Regarding the covariates, age, BMI, years of radiological service, daily working hours, and sleep duration were treated as continuous variables to maximize statistical power and minimize information loss. Other categorical covariates, such as education background, academic title, and lifestyle factors, were incorporated into the multivariable models using dummy coding.

## Results

### Baseline information of included participants

This cross-sectional study comprised 1,080 occupational radiation workers from Guangzhou. The cohort had a mean age of 38.7 ± 9 years, with individuals in the morbidity group being significantly older (40.8 ± 9.2 years) than those without morbidity (36.9 ± 8.5 years). Females constituted a higher proportion (59.4%) of participants. The average BMI was 23.0 ± 3.1. There was no statistically significant difference in BMI between those with and without morbidity. Participants had an average of 11.1 ± 8.8 years of radiological service, with significantly longer exposure durations observed in the morbidity group (12.7 ± 9.4 vs. 9.7 ± 8.1 years). Educational backgrounds varied, with 59.2% holding a bachelor’s degree or below, while 11.1% held doctoral degrees. Clinical healthcare roles predominated (66.9%), followed by biomedical engineering (33.1%). Lifestyle factors revealed that 49.1% currently consumed alcohol, and 7.4% were current smokers. Physical exercise frequency differed significantly between groups, with 67.9% reporting 1–3 sessions per week. Daily sleep duration also varied, as 78.9% slept 6 ~ 8 h per night, while 16.6% slept ≤6 h (Table 1).

**Table 1:** Baseline information of included participants.

Variables	Overall (n = 1,080)	With morbidity (n = 491)	Without morbidity (n = 589)	*p*-value
Age/years	38.7 ± 9	40.8 ± 9.2	36.9 ± 8.5	<0.001
Sex				<0.001
Male	439(40.6)	213(43.4)	226(38.4)	
Female	641(59.4)	278(56.6)	363(61.6)	
Race				0.666
Han	1,058(98)	480(97.8)	578(98.1)	
Other	22(2)	11(2.2)	11(1.9)	
Height/cm	166.7 ± 8.1	166.3 ± 7.7	167.2 ± 8.3	<0.001
Weight/kg	64.1 ± 11.4	64.3 ± 11.4	64 ± 11.5	0.674
BMI	23 ± 3.1	23.1 ± 3	22.8 ± 3.2	0.082
Years of radiological service/years	11.1 ± 8.8	12.7 ± 9.4	9.7 ± 8.1	<0.001
Daily working hours/h	7.8 ± 1.8	7.8 ± 1.7	7.8 ± 1.9	0.955
Education background				<0.001
Bachelor’s degree and below	639(59.2)	316(64.4)	323(54.8)	
Master’s degree	321(29.7)	129(26.3)	192(32.6)	
Doctor of philosophy	120(11.1)	46(9.4)	74(12.6)	
Professions				0.830
Clinical healthcare	722(66.9)	330(67.2)	392(66.6)	
Biomedical engineering	358(33.1)	161(32.8)	197(33.4)	
Position responsibilities				<0.001
Diagnostic Radiology	520(48.1)	225(45.8)	295(50.1)	
Interventional Radiology	300(27.8)	133(27.1)	167(28.4)	
Radiation Therapy	103(9.5)	47(9.6)	56(9.5)	
Nuclear Medicine	68(6.3)	36(7.3)	32(5.4)	
Others	89(8.2)	50(10.2)	39(6.6)	
Academic title				<0.001
Junior	376(34.8)	146(29.7)	230(39)	
Intermediate	452(41.9)	222(45.2)	230(39)	
Senior	252(23.3)	123(25.1)	129(21.9)	
In-room operation	353(32.7)	165(33.6)	188(31.9)	0.559
Active Smoking Status				0.652
Never smoked	975(90.3)	441(89.8)	534(90.7)	
Have quit smoking	25(2.3)	13(2.6)	12(2)	
Currently smoke	80(7.4)	37(7.5)	43(7.3)	
Alcohol Consumption Status				<0.001
Never drink alcohol	537(49.7)	224(45.6)	313(53.1)	
Have quit drinking	13(1.2)	10(2)	3(0.5)	
Currently drink alcohol	530(49.1)	257(52.3)	273(46.3)	
Physical Exercise Status				0.022
Never exercise	196(18.1)	91(18.5)	105(17.8)	
1 ~ 3 times/week	733(67.9)	317(64.6)	416(70.6)	
4 ~ 6 times/week	114(10.6)	60(12.2)	54(9.2)	
Daily exercise	37(3.4)	23(4.7)	14(2.4)	
Daily Sedentary Time				0.167
<2 h	127(11.8)	67(13.6)	60(10.2)	
2 ~ 4 h	393(36.4)	178(36.3)	215(36.5)	
>4 h	560(51.9)	246(50.1)	314(53.3)	
Frequency of staying up late				0.913
Never	205(19)	91(18.5)	114(19.4)	
1 ~ 2 times/week	587(54.4)	267(54.4)	320(54.3)	
3 ~ 6 times/week	204(18.9)	95(19.3)	109(18.5)	
Daily	84(7.8)	38(7.7)	46(7.8)	
Daily Sleep Duration				0.043
≤6 h	179(16.6)	91(18.5)	88(14.9)	
6 ~ 8 h	852(78.9)	384(78.2)	468(79.5)	
≥8 h	49(4.5)	16(3.3)	33(5.6)	

### Prevalence of morbidity among occupational radiation workers

**Figure 2:**
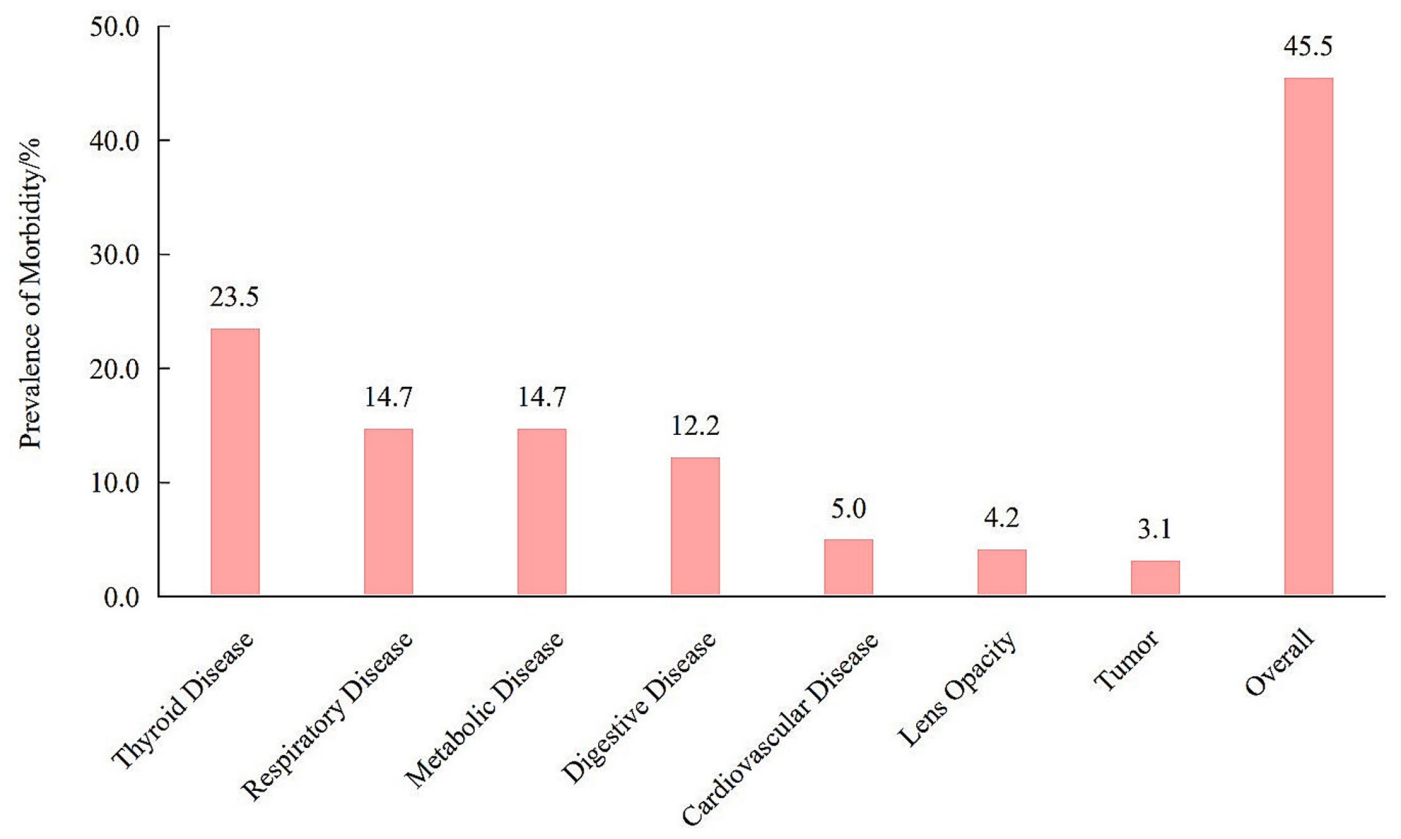
Prevalence of morbidity among occupational radiation workers.

The overall morbidity prevalence in this occupational cohort was 45.5%. Thyroid disease constituted the most common morbidity, with a prevalence of 23.5%. Respiratory and metabolic diseases exhibited an equal prevalence of 14.7%, while digestive disease followed at 12.2%. The prevalence of cardiovascular disease, lens opacity, and tumors was comparatively lower, recorded at 5.0, 4.2, and 3.1%, respectively (Figure 2).

### Dietary factors by morbidity status

Significant differences in dietary habits were observed between participants with and without morbidity. Tea consumption frequency showed a distinct distribution, with 27.1% of the morbidity group consuming tea ≥3 times/week compared to 31.9% in the non-morbidity group (*p* = 0.041). Red meat intake was notably higher in the morbidity group, with 86.8% consuming it ≥3 times/week versus 79.3% in the non-morbidity group (*p* = 0.001). Conversely, vegetable consumption ≥3 times/week was lower in the morbidity group (85.1%) compared to the non-morbidity group (92.7%, *p* < 0.001). Fruit intake ≥200 g/week was also less frequent among those with morbidity (70.9% vs. 76.4%, *p* = 0.040). Food preference patterns differed, with a higher proportion of participants in the morbidity group favoring meat (22.2% vs. 18.8%) and a lower proportion preferring vegetables (3.7% vs. 7.1%, *p* = 0.048) (Table 2).

**Table 2:** Comparison of dietary factors between occupational radiation workers with and without morbidity.

Variables	Term	With morbidity		Without morbidity		*p*-value
		*n*	%	*n*	%	
Tea	Never	67	13.6	53	9.0	0.041
	1 ~ 2 times/week	291	59.3	348	59.1	
	≥3 times/week	133	27.1	188	31.9	
Coffee	Never	139	28.3	151	25.6	0.647
	1 ~ 2 times/week	273	55.6	339	57.6	
	≥3 times/week	79	16.1	99	16.8	
Seafood	0 ~ 2 times/week	461	93.9	547	92.9	0.503
	≥3 times/week	30	6.1	42	7.1	
Red meat	0 ~ 2 times/week	65	13.2	122	20.7	0.001
	≥3 times/week	426	86.8	467	79.3	
Vegetable	0 ~ 2 times/week	73	14.9	43	7.3	<0.001
	≥3 times/week	418	85.1	546	92.7	
Fruit	0 ~ 199 g/week	143	29.1	139	23.6	0.040
	≥200 g/week	348	70.9	450	76.4	
Taste preferences	Normal	413	84.1	495	84.0	0.786
	Prefer spicy	10	2.0	8	1.4	
	Prefer sour	68	13.8	86	14.6	
Food preferences	Balanced nutrition	364	74.1	436	74.0	0.048
	Prefer meat	109	22.2	111	18.8	
	Prefer vegetable	18	3.7	42	7.1	

### Multivariable logistic regression analysis

Multivariable logistic regression analysis was performed to evaluate the independent associations between dietary factors and morbidity burden after adjusting for confounding variables. In the fully adjusted model (Model 2), frequent tea consumption was significantly associated with lower odds of morbidity, with participants consuming tea ≥3 times/week exhibiting 59% lower morbidity odds (OR 0.41, 95% CI 0.26 ~ 0.64) compared to non-tea drinkers. Conversely, high red meat intake (≥3 times/week) as compared to 0 ~ 2 times/week was associated with substantially increased morbidity odds (OR 1.88, 95% CI 1.34 ~ 2.67). Vegetable consumption ≥3 times/week (OR 0.44, 95% CI 0.29 ~ 0.67) as compared to 0 ~ 2 times/week and fruit intake ≥200 g/week (OR 0.71, 95% CI 0.53 ~ 0.95) as compared to 0 ~ 199 g/week both showed inverse associations with morbidity. Dietary preferences were also significantly associated with outcomes, as meat-preferring individuals as compared to those preferring balanced nutrition had elevated morbidity odds (OR 1.44, 95% CI 1.05 ~ 1.99), while vegetable-preferring participants compared to the balanced nutrition group showed reduced odds (OR 0.42, 95% CI 0.22 ~ 0.75). Coffee consumption, seafood intake, and taste preferences did not demonstrate statistically significant associations in the final model (Table 3).

**Table 3:** Multivariable logistic regression analysis of dietary factors and morbidity burden in occupational radiation workers.

Variables	Term	Crude model			Model 1			Model 2		
		OR	95%CI	*p*-value	OR	95%CI	*p*-value	OR	95%CI	*p*-value
Tea	Never	Ref.			Ref.			Ref.		
	1 ~ 2 times/week	0.66	0.45 ~ 0.98	0.039	0.64	0.43 ~ 0.95	0.029	0.55	0.36 ~ 0.82	0.004
	≥3 times/week	0.56	0.37 ~ 0.85	0.007	0.49	0.31 ~ 0.75	0.001	0.41	0.26 ~ 0.64	<0.001
Coffee	Never	Ref.			Ref.			Ref.		
	1 ~ 2 times/week	0.87	0.66 ~ 1.16	0.349	0.93	0.70 ~ 1.25	0.64	0.88	0.65 ~ 1.18	0.389
	≥3 times/week	0.87	0.60 ~ 1.26	0.455	0.97	0.66 ~ 1.43	0.891	0.97	0.66 ~ 1.45	0.897
Seafood	0 ~ 2 times/week	Ref.			Ref.			Ref.		
	≥3 times/week	0.85	0.52 ~ 1.37	0.503	0.71	0.43 ~ 1.16	0.173	0.73	0.44 ~ 1.21	0.228
Red meat	0 ~ 2 times/week	Ref.			Ref.			Ref.		
	≥3 times/week	1.71	1.24 ~ 2.39	0.001	1.82	1.31 ~ 2.57	<0.001	1.88	1.34 ~ 2.67	<0.001
Vegetable	0 ~ 2 times/week	Ref.			Ref.			Ref.		
	≥3 times/week	0.45	0.30 ~ 0.67	<0.001	0.46	0.30 ~ 0.69	<0.001	0.44	0.29 ~ 0.67	<0.001
Fruit	0 ~ 199 g/week	Ref.			Ref.			Ref.		
	≥200 g/week	0.75	0.57 ~ 0.99	0.040	0.74	0.56 ~ 0.97	0.032	0.71	0.53 ~ 0.95	0.020
Taste preferences	Normal	Ref.			Ref.			Ref.		
	Prefer spicy	0.95	0.67 ~ 1.34	0.759	1.11	0.77 ~ 1.58	0.576	1.07	0.75 ~ 1.54	0.704
	Prefer sour	1.50	0.59 ~ 3.96	0.399	1.74	0.67 ~ 4.66	0.256	1.67	0.64 ~ 4.53	0.297
Food preferences	Balanced nutrition	Ref.			Ref.			Ref.		
	Prefer meat	1.18	0.87 ~ 1.59	0.287	1.44	1.05 ~ 1.97	0.022	1.44	1.05 ~ 1.99	0.026
	Prefer vegetable	0.51	0.28 ~ 0.89	0.022	0.46	0.25 ~ 0.82	0.010	0.42	0.22 ~ 0.75	0.004

Footprint. Model 1 was adjusted for sex and age. Model 2 was adjusted for multiply variables including sex, age, race, BMI, years of radiological service, daily working, education background, professions, position responsibilities, academic title, in-room operation, active smoking status, alcohol consumption status, physical exercise status, daily sedentary time, frequency of staying up late and sleep duration.

## Discussion

This cross-sectional study identified significant associations between specific dietary factors and morbidity burden among occupational radiation workers in Guangzhou, China. The findings suggest that higher consumption of tea, vegetables, and fruits is associated with reduced odds of chronic morbidity, whereas frequent red meat intake and a preference for meat over vegetables are linked to higher morbidity odds. These observations align with the hypothesis that dietary patterns rich in plant-based foods and bioactive compounds may be linked to the health status of chronic low-dose radiation exposure (20).

### Interpretation of key findings

The inverse association between tea consumption (≥3 times/week) and morbidity burden (OR 0.41) is consistent with experimental and epidemiological evidence on the radioprotective and antioxidant properties of tea polyphenols (20, 21). *In vitro* and animal studies demonstrate that catechins, particularly epigallocatechin gallate, attenuate radiation-induced DNA damage, reduce oxidative stress markers, and enhance DNA repair capacity (21, 22). Population-based studies in occupational cohorts have similarly reported that regular tea intake is associated with lower risks of cardiovascular and metabolic disorders (20), which are prominent components of the morbidity spectrum observed in this cohort. The absence of a significant association with coffee consumption in the present analysis further supports the specificity of tea as a potential protective factor in this context, possibly due to its unique polyphenol profile and cultural consumption patterns in southern China.

The inverse associations of vegetable (OR = 0.44) and fruit intake (OR = 0.71) against morbidity burden are biologically plausible given the multifaceted roles of plant-based foods in mitigating oxidative stress and inflammation (23, 24). Although the effect sizes were moderate after merging the lower intake categories as the reference group, the associations remained statistically significant and directionally consistent with prior evidence. Fruits and vegetables are rich sources of vitamins C and E, carotenoids, and flavonoids, which scavenge free radicals, modulate inflammatory pathways, and support endothelial function (23, 25). Chronic radiation exposure is known to deplete endogenous antioxidants and promote lipid peroxidation, thereby exacerbating metabolic and cardiovascular conditions (26). The high prevalence of thyroid diseases (23.5%) in this cohort underscores the susceptibility of endocrine tissues to radiation-induced oxidative damage. Experimental data indicate that dietary antioxidants can reduce radiation-induced thyroid dysfunction by preserving thyrocyte integrity and modulating autoimmune responses (27). The observed associations may thus reflect a partial attenuation of radiation-induced metabolic and endocrine perturbations through enhanced antioxidant and anti-inflammatory capacity.

Conversely, the positive association between high red meat intake (≥3 times/week) and morbidity burden (OR = 1.88) is consistent with extensive literature linking red meat consumption to inflammation, insulin resistance, and cardiovascular disease (28, 29). Mechanistically, heme iron, saturated fats, and advanced glycation end-products in red meat promote oxidative stress, endothelial dysfunction, and systemic inflammation—pathways that may synergize with radiation-induced metabolic dysregulation (28, 30). The preference for meat over vegetables further amplifies these risks by displacing protective plant-based foods, as evidenced by the elevated morbidity odds among meat-preferring individuals (OR = 1.44) and the reduced odds among those preferring vegetables (OR = 0.42). This pattern aligns with global dietary transitions characterized by increased meat consumption and declining plant food intake, which have been implicated in the rising burden of chronic diseases in China (31).

### Contextualization within occupational radiation health

Occupational radiation workers represent a unique population with cumulative low-dose exposure, for whom traditional radioprotective strategies primarily focus on physical shielding and dose monitoring. The present findings suggest that dietary factors may serve as modifiable determinants of health outcomes in this group, although it remains unclear to what extent these associations are specific to the occupational environment compared to the general public. The morbidity burden of 45.5% observed here is substantial, with thyroid diseases being the most prevalent condition. This pattern resonates with studies reporting elevated risks of thyroid dysfunction and autoimmune thyroiditis among medical radiation workers, particularly those with longer service durations (27, 32). The association between longer radiological service (mean 12.7 years in the morbidity group) and higher morbidity odds underscores the cumulative nature of radiation-related health effects, which may be exacerbated by suboptimal dietary patterns.

The protective associations of plant-based foods and tea are particularly relevant given the mechanistic overlap between radiation-induced damage and the pathophysiology of chronic diseases. Ionizing radiation generates reactive oxygen species (ROS), leading to DNA strand breaks, lipid peroxidation, and chronic low-grade inflammation (26, 33). Nutrients abundant in fruits, vegetables, and tea can directly quench ROS, enhance glutathione synthesis, and upregulate antioxidant enzymes such as superoxide dismutase and catalase (23, 25). Additionally, dietary fiber and phytochemicals modulate gut microbiota composition, which in turn influences systemic inflammation and metabolic health—a pathway that may be especially important in radiation workers given emerging evidence on radiation-induced gut dysbiosis (34).

### Comparison with existing literature

The benefits of plant-based diets against chronic diseases are well-established in general populations, but evidence specific to occupational radiation workers is limited (24, 31). A recent cohort study of nuclear industry workers reported that adherence to a Mediterranean-style diet was associated with lower all-cause mortality and reduced cardiovascular events, independent of radiation dose (24). Similarly, studies among astronauts and radiation-exposed populations have highlighted the potential of antioxidant-rich diets to mitigate oxidative stress and preserve immune function (25). The present study extends these observations by focusing on a composite morbidity outcome and identifying specific food groups (tea, vegetables, fruits, red meat) that contribute to disease burden in a real-world occupational setting.

The associations observed here remain meaningful after merging lower-frequency intake categories as the reference group, with OR values ranging from 0.41 to 1.88 across dietary factors. These effect sizes are comparable to those reported in dietary interventions targeting cardiovascular risk reduction. For instance, meta-analyses of randomized trials have shown that increased fruit and vegetable consumption reduces systolic blood pressure by 2–4 mmHg and LDL cholesterol by 0.1 ~ 0.2 mmol/L, translating to approximately 10 ~ 20% reductions in cardiovascular event risk (23). The stronger associations in the fully adjusted model (Model 2) suggest that the dietary associations are independent of key confounders such as age, sex, BMI, occupational factors, and lifestyle behaviors.

### Methodological considerations and limitations

Several limitations should be acknowledged. First, the cross-sectional design precludes causal inference, and reverse causality—where individuals with morbidity alter their diet—cannot be ruled out. Second, dietary assessment via a simplified FFQ is subject to recall bias and measurement error; specifically, the lack of detailed portion sizes prevented the calculation of total energy intake, although we adjusted for BMI and physical activity as proxy indicators of energy balance. Third, the dietary frequency categories were relatively broad and predefined by the original survey, which limited post-hoc refinement. To address the imbalance in sample sizes across intake categories, the two lowest frequency groups for seafood, red meat, vegetables, and fruit were merged into a single reference category, which improved the stability of the logistic regression estimates but should be noted when interpreting the findings. Fourth, the “Healthy Worker Effect” may introduce selection bias, as individuals who left the workforce due to severe illness were not captured, potentially leading to an underestimation of morbidity prevalence and an attenuation of the observed associations. Finally, although we adjusted for various demographic and occupational factors, residual confounding from factors like precise radiation dose metrics or genetic predisposition remains possible. Future longitudinal studies with non-exposed controls and more granular dietary records are warranted to confirm these findings.

### Implications for practice and future research

From a public health perspective, these findings suggest that dietary interventions could complement existing radioprotective measures for occupational radiation workers. Integrating tailored nutritional guidelines into occupational health frameworks may help mitigate the long-term health risks associated with chronic low-dose exposure. Practical recommendations could include promoting daily consumption of fruits and vegetables (≥200 g/day), encouraging regular tea intake (particularly green tea), and limiting red meat to ≤1 ~ 2 servings per week. Such strategies align with national dietary guidelines in China, which emphasize plant-based diets and reduced meat consumption to address the dual burden of malnutrition and chronic diseases (31).

Future research should prioritize longitudinal studies with repeated dietary assessments and objective biomarkers of oxidative stress and inflammation to establish temporal relationships and elucidate underlying mechanisms (33, 34). Intervention trials testing the efficacy of plant-based dietary patterns or specific bioactive compounds (e.g., tea polyphenols) in reducing morbidity endpoints among radiation workers would provide stronger evidence for causal relationships. Additionally, exploring gene-diet interactions may identify subgroups with heightened susceptibility to radiation-related harm who could benefit most from targeted nutritional interventions.

## Conclusion

In summary, this study provides evidence that dietary factors—particularly tea, vegetables, and fruit intake—are independently and inversely associated with morbidity burden among occupational radiation workers, while frequent red meat consumption confers higher odds. These findings support integrating nutrition-focused strategies into occupational health programs, pending confirmation from future longitudinal studies.

## Data Availability

The raw data supporting the conclusions of this article will be made available by the authors, without undue reservation.
