# Supplementary material for: Associations between dietary factors and morbidity burden in occupational radiation workers: a cross-sectional study in Guangzhou, China
Source: Front Nutr. 2026 Jul 31;13:1808021. doi: 10.3389/fnut.2026.1808021 (PMC13472953; doi:10.3389/fnut.2026.1808021)
Supplement: Supplementary file 1 [file Table_1.docx]

**Occupational Health Survey for Radiation Workers**

Please answer the following questions carefully based on your actual situation. Fill in the blanks and tick (√) your best answer in the options.

1.Name of Work Unit: _______________ Full Name: _______________

2.Type of Radiation Work: □ Diagnostic Radiology □ Dental Radiology □ Nuclear Medicine □ Radiation Therapy □ Interventional Radiology □ Others (___________)

3.Job Position: □ Physician □ Technician □ Nurse □ Physicist □ Chemist □ Engineer □ Others (___________)

4.Length of Service in Radiation Work: ____ years; Year of starting current radiation work: ____

5.Gender: □ Male □ Female

6.ID Number: _______________

7.Address: ____ Province ____ City ____ District/County

8.Ethnicity: □ Han □ Ethnic Minority (___________)

9.Height: ____ cm Weight: ____ kg Waist Circumference: ____ cm Abdominal Circumference: ____ cm Hip Circumference: ____ cm

10.Professional Title: □ Junior & Below □ Intermediate □ Senior

11.Education: □ Associate Degree & Below □ Bachelor’s Degree □ Master’s Degree □ Doctoral Degree

12.Daily Working Hours: ____ hours

13.Do you handle Iodine-131? □ No □ Yes;

Do you handle Technetium-99m? □ No □ Yes;

Do you handle Fluorine-18? □ No □ Yes

14.Have you ever performed in-room operations (e.g., in-room fluoroscopy/radiography, orthopedic reduction beside equipment)? □ No □ Yes

15.Effect of Regular Radiation Protection Training: □ Very Effective □ Somewhat Effective □ Not Effective □ Not Attended Training

16.Active Smoking Status: □ Never Smoked □ Quit Smoking (____ years) □ Current Smoker (□ 1–5 cigarettes/day □ 6–10 cigarettes/day □ 11–20 cigarettes/day □ >20 cigarettes/day)

17.Alcohol Consumption: □ Never Drank □ Quit Drinking (____ years) □ Occasionally (<2 times/week) □ Frequently (≥2 times/week, □ ≤100 ml/week □ >100 ml/week)

18.Tea Drinking: □ Never □ Occasionally □ ≤5 days/week □ >5 days/week

19.Coffee Drinking: □ Never □ Occasionally □ ≤5 days/week □ >5 days/week

20.Dietary Habits (Multiple Choice): Taste (□ Normal □ Spicy □ Sour □ Sweet); Meat & Vegetable Preference (□ Meat-Heavy □ Vegetable-Heavy □ Balanced Diet)

21.Seafood Intake (kelp, laver, shellfish, fresh marine fish, etc.): □ None □ <3 times/week □ ≥3 times/week

22.Meat Intake: □ None □ <3 times/week □ ≥3 times/week

23.Vegetable Intake: □ None □ <3 times/week □ ≥3 times/week

24.Fruit Intake: □ None □ <200 g/week □ ≥200 g/week

25.Physical Activity (≥30 minutes per session): □ Never □ 1–3 times/week □ 3–6 times/week □ Daily

26.Sedentary Time: □ ≤2 hours/day □ 2–4 hours/day □ ≥4 hours/day

27.Sleep Duration: □ ≤6 hours/day □ 6–8 hours/day □ ≥8 hours/day

28.Sleep Disorders (Multiple Choice): □ None □ Difficulty Falling Asleep □ Difficulty Maintaining Sleep □ Early Awakening □ Non-Restorative Sleep □ Daytime Sleepiness

29.Staying Up Late (falling asleep after 00:00): □ Never □ 1–3 times/week □ ≥3 times/week □ Every Night

30.Current/Past Medical History:

Ever had cancer (current or past): □ No □ Yes, Cancer Name: _______________, Year of Diagnosis: ____

31.Persistently low white blood cell count (<3.5×10⁹/L for >6 months): □ No □ Yes, Year of Onset: ____

32.Thyroid Diseases: □ None □ Hyperthyroidism □ Thyroid Nodules □ Goiter □ Thyroiditis □ Hypothyroidism □ Thyroid Adenoma □ Thyroid Cancer; Year of Diagnosis: ____

33.Lens Opacity: □ No □ Yes, Year of Diagnosis: ____

34.Radiation-induced Skin Injury: □ No □ Yes, Year of Diagnosis: ____

35.Cardiovascular & Cerebrovascular Diseases: □ None □ Hypertension □ Arrhythmia □ Coronary Heart Disease □ Stroke □ Others (___________); Year of Diagnosis: ____

36.Metabolic Diseases: □ None □ Hyperlipidemia □ Diabetes □ Hyperuricemia □ Fatty Liver □ Others (___________); Year of Diagnosis: ____

37.Digestive Diseases: □ None □ Chronic Enteritis □ Chronic Gastritis □ Peptic Ulcer □ Cholecystitis □ Gastroesophageal Reflux □ Gastric Cancer □ Liver Cancer □ Colorectal Cancer □ Others (___________); Year of Diagnosis: ____

38.Respiratory Diseases: □ None □ Rhinitis □ Pharyngitis □ Bronchiectasis □ Bronchial Asthma □ Lung Cancer □ Others (___________); Year of Diagnosis: ____

39.Skin Rashes: □ None □ Dry & Chapped Skin □ Eczema □ Contact Dermatitis □ Others (___________); Year of Diagnosis: ____

40.Others (Please specify): _______________
